# Yeast genetic interaction screens in the age of CRISPR/Cas

**DOI:** 10.1007/s00294-018-0887-8

**Published:** 2018-09-25

**Authors:** Neil R. Adames, Jenna E. Gallegos, Jean Peccoud

**Affiliations:** Department of Chemical and Biological Engineering, Colorado State University, Fort Collins, CO 80523 USA

**Keywords:** Genetic interaction screens, Synthetic genetic array, CRISPR/Cas9, Functional genomics, dSLAM, Green Monster, *Saccharomyces cerevisiae*

## Abstract

The ease of performing both forward and reverse genetics in *Saccharomyces cerevisiae*, along with its stable haploid state and short generation times, has made this budding yeast the consummate model eukaryote for genetics. The major advantage of using budding yeast for reverse genetics is this organism’s highly efficient homology-directed repair, allowing for precise genome editing simply by introducing DNA with homology to the chromosomal target. Although plasmid- and PCR-based genome editing tools are quite efficient, they depend on rare spontaneous DNA breaks near the target sequence. Consequently, they can generate only one genomic edit at a time, and the edit must be associated with a selectable marker. However, CRISPR/Cas technology is efficient enough to permit markerless and multiplexed edits in a single step. These features have made CRISPR/Cas popular for yeast strain engineering in synthetic biology and metabolic engineering applications, but it has not been widely employed for genetic screens. In this review, we critically examine different methods to generate multi-mutant strains in systematic genetic interaction screens and discuss the potential of CRISPR/Cas to supplement or improve on these methods.

## Introduction

The budding yeast *Saccharomyces cerevisiae* has been a workhorse model organism for genetics for over 50 years now. One of the major advances in yeast genetics was the generation of loss-of-function (LOF)-mutant strain collections by systematically deleting all non-essential annotated open reading frames (Winzeler et al. 1999). These collections are derived from a common set of background strains and include haploid strains, homozygous diploid strains, and heterozygous diploid strains. To supplement these collections, groups have also generated collections of strains (in the same genetic background) carrying temperature-sensitive, transcriptionally repressible, or hypomorphic alleles of essential genes (Mnaimneh et al. 2004; Breslow et al. 2008; Li et al. 2011; Wang et al. 2015; Kofoed et al. 2015).

While functional analyses of single-gene perturbations are useful, by combining LOF mutations in the same cell, one can uncover suppressing or enhancing epistatic interactions that provide clues about the network structures of cellular pathways (Salminen and Novick 1987; Adams et al. 1990, 1993; Bendert and Pringle 1991; Costigan et al. 1992; Frank et al. 1992; Scidmore et al. 1993). There are two types of genetic interactions (GIs) that can occur when two or more LOF mutations are combined in the same cell–synthetic suppression or synthetic enhancement. An extreme example of synthetic suppression is synthetic rescue, when one or more of the single mutants is inviable, but the synthetic combination is viable. An extreme example of synthetic enhancement is synthetic lethality, when all single mutants are viable, but the synthetic combination is lethal.

Gain-of-function (GOF) mutations are routinely generated by gene overexpression (dosage) from a plasmid or an integrated construct using a strong promoter. Dosage screens in yeast are largely successful because there is little dosage compensation at the single-gene level (Deutschbauer et al. 2005; Springer et al. 2010; Ishikawa et al. 2017).

LOF mutations can also be combined with GOF alleles in the same cell and assessed for dosage interactions. The extreme case of dosage suppression is dosage rescue of an otherwise lethal mutation. The extreme case of dosage enhancement is dosage lethality, in which neither overexpression of gene *x* nor the mutation of gene *y* are lethal, but the combination is. One can also overexpress two genes to find double dosage lethality, but this is not a common approach (Youn et al. 2017).

The goal of these mutant combinations is to identify epistatic GIs from which one can infer pathway hierarchies (Forsburg 2001; Boone et al. 2007; Costanzo et al. 2010; Magtanong et al. 2011). For example, synthetic suppression and dosage enhancement are both consistent with a network in which the two gene products are functionally antagonistic. In contrast, synthetic enhancement and dosage suppression are both consistent with gene products that work together or in parallel in the same cellular process.

Traditionally, GIs were discovered by random mutagenesis of LOF or GOF mutant query strains, or by transforming such strains with high copy number cDNA or genomic DNA plasmid libraries, followed by a selection regimen or visual screen to find suppressing or enhancing interactions (Reed et al. 1989; Albertini and Zimmermann 1991; Costigan et al. 1992; Chowdhury et al. 1992; Flescher et al. 1993; Puziss et al. 1994; Blázquez and Gancedo 1994; Machin et al. 1995; Kroll et al. 1996; Kaytor and Livingston 1996; Akada et al. 1997; Mullen et al. 2001; Stevenson et al. 2001; Kitazono and Kron 2002; Bogomolnaya et al. 2004; Kaplan and Kupiec 2007; Carlsson et al. 2018). Although the process of screening using mutagenesis or pooled plasmid libraries is relatively quick and easy, the resulting strains require extensive characterization to ensure screen “saturation” (i.e., obtaining multiple alleles of the same genes), and to identify the genes responsible for the interactions.

Now, GI screens are routinely performed using systematic arrayed yeast strain collections or arrayed plasmid collections, which ensure comprehensive genomic coverage. There are several approaches researchers have taken to perform such screens—transformation of a systematic collection of overexpression (OE) plasmids into one or a few mutant query strains, transformation of one or a few query plasmids into a systematic collection of yeast mutant strains, or crossing one or a few query mutant strains to a systematic collection of mutant strains and obtaining haploid double mutants. Although such screens require complex high-throughput protocols up front, the back-end analysis and identification of the interacting genes is much faster and easier than in traditional GI screens.

In the following sections, we will examine and compare traditional methods to perform systematic GI screens and discuss the advantages and limitations of each approach. These approaches combine mutations either by crossing mutant strains, or by introducing a common mutation into a collection of pre-existing mutants. In both approaches, query mutations are introduced into cells by homology-directed repair (HDR) with transformed donor DNA [for a comprehensive review of how HDR works, see (Gallagher and Haber 2018)]. Chromosomal recombination with the donor DNA depends on the spontaneous generation of double-strand breaks (DSBs) at the target locus. These methods require a selectable marker in the donor DNA to select for the rare recombinants and are also too inefficient to perform more than one type of edit at a time (multiplexing). Sequence-specific endonucleases improve editing efficiency enough to allow markerless and multiplexed edits (Guha and Edgell 2017). CRISPR/Cas is the most popular method of endonuclease-mediated gene editing because of the ease of programming the target specificity. We will discuss how CRISPR/Cas has the potential to complement and streamline current methods of performing GI screens. Our hope is that this review will help yeast geneticists to make informed decisions about what approaches best suit their particular genetic screens.

## Traditional genetic interaction screens

### Overexpression plasmid transformation

The simplest approach to dosage interaction screens is to transform overexpression (OE) plasmids into mutant strains. These screens can be performed systematically using the genomic collections of yeast strains we mentioned earlier or genomic collections of OE plasmids.

**Fig. 1:**
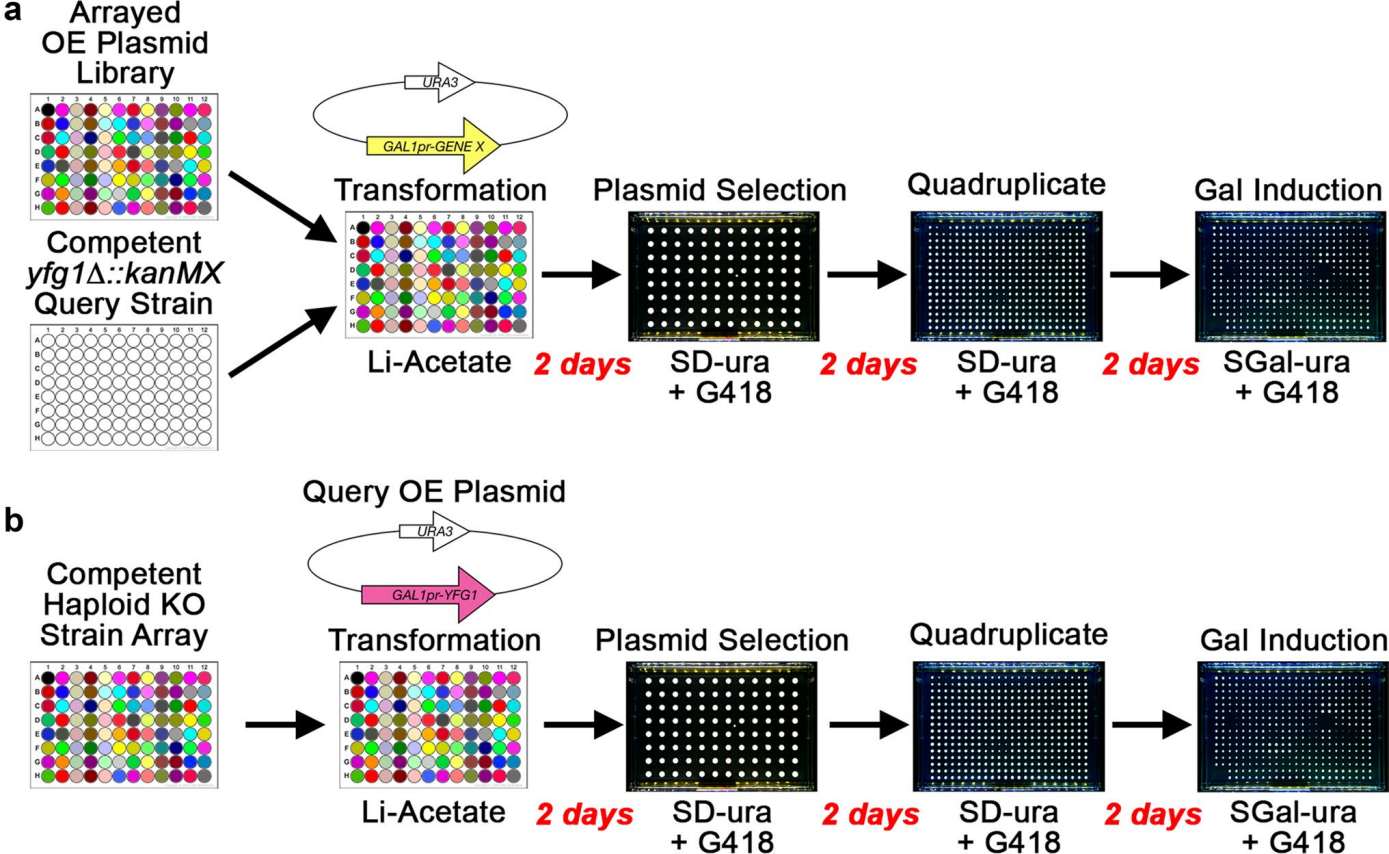
Dosage GI screens using OE plasmids. **a** The dosage interaction screen starts with two 96-well plates. One plate contains an arrayed OE plasmid library as DNA, each well overexpressing a different single ORF. Every well in the second plate contains the same query mutant strain denoted by your favorite gene *yfg1∆*. **b** The dosage interaction screen starts with a query overexpression plasmid and a 96-well plate containing an array of haploid deletion strains, each well containing a different deletion strain. Transformations are performed in liquid in 96-well plates. Transformants may undergo selection in liquid by transferring cells into fresh plates or by replica pinning to agar selection plates (shown). In this example, selection of the plasmid is maintained by growth on media lacking uracil (SD-ura), and selection of the gene deletions is maintained by adding G418 as the deletion collections carry *kanMX*. Transformants are assayed for growth under non-induced (SD-ura) and induced (SGal-ura) conditions. Transformants should be quadruplicated to 384 arrays for statistical measurements of growth. Plates are imaged and colony sizes are measured to determine if two genes interact. Red text indicates how many days are required for each step in the workflow. *kanMX*: G418-resistance marker, *OE*: overexpression, *yfg1∆*: deletion of your favorite gene, *Gal*: galactose; colored wells indicate that the contents of each well are different

To introduce plasmids into large arrays of different yeast strains, or to introduce large arrays of different plasmids into the same yeast strain, transformations can be readily performed in 96-well plates to accommodate 4000 or more strains or plasmids (Fleming and Gitler 2011) (Fig. 1).

Using plasmid transformation, systematic GI screens have been performed in two different ways. First, one could transform query mutants with systematic arrays of OE plasmids to find synthetic dosage enhancement or lethality interactions (Zimmermann et al. 2017), or to find dosage suppression or rescue interactions (Magtanong et al. 2011; Patra et al. 2017) (Fig. 1a). The alternative approach is to transform a query OE plasmid into a systematic mutant strain collection. We do not know of any examples in which this approach was performed to find synthetic dosage interactions using the entire yeast deletion collection, but it has been done with a collection of mutants (Kroll et al. 1996) (Fig. 1b). One could also transform a query OE plasmid into a subset of the haploid deletion strains that show low fitness, or into hypomorphic/conditional essential gene collections to find dosage suppression interactions.

Available systematic collections of plasmids for dosage screens have been constructed by inserting PCR-amplified ORFs behind a strongly galactose-inducible *GAL1* promoter (Zhu et al. 2001; Gelperin et al. 2005; Hu et al. 2007), or by cloning the entire PCR-amplified gene, including its natural promoter and terminator, into a 2 µ plasmid (Moriya et al. 2006; Magtanong et al. 2011). 2 µ plasmids use an origin of replication that replicates independently of the host’s cell cycle. These plasmids replicate to high copy number, but because they do not have centromeres, they randomly segregate to daughter cells and are present in the population of cells at a wide range of copy numbers. Both the inducible and 2 µ plasmids are maintained as episomal circular DNA.

When performing a systematic search for negative GIs such as synthetic dosage lethality, high copy number plasmids should be avoided. The reason for this is that a lack of growth of the transformants could be due to synthetic dosage lethality or simply a technical failure of the transformation. Using an inducible overexpression system avoids this problem because transformants can be selected under uninduced conditions before exposing them to overexpression conditions.

In addition, about 20% of genes can have negative effects on cellular growth on their own (Makanae et al. 2013). One way to study synthetic dosage interactions with these genes is to control the level of their expression. When using the inducible OE plasmid collections, there is little ability to tune the level of overexpression because the *GAL1* promoter used to drive gene overexpression responds to the presence of galactose in the medium in a switch-like manner (Hawkins and Smolke 2006). Some overexpression genomic DNA libraries and systematic collections use a 2 µ plasmid carrying the *URA3* selection marker and a second marker consisting of a partial loss-of-function *leu2-89* (*LEU2-d*) allele (Moriya et al. 2006; Carlsson et al. 2018). Growth of the transformants on media lacking uracil alone allows cells to maintain a relatively low average copy number, but restricting or removing leucine in the medium selects for cells with higher copy numbers to allow sufficient production of leucine from the weakened Leu2-89 enzyme. Therefore, the copy number and gene dosage can be tuned with the concentration of leucine in the medium.

**Fig. 2:**
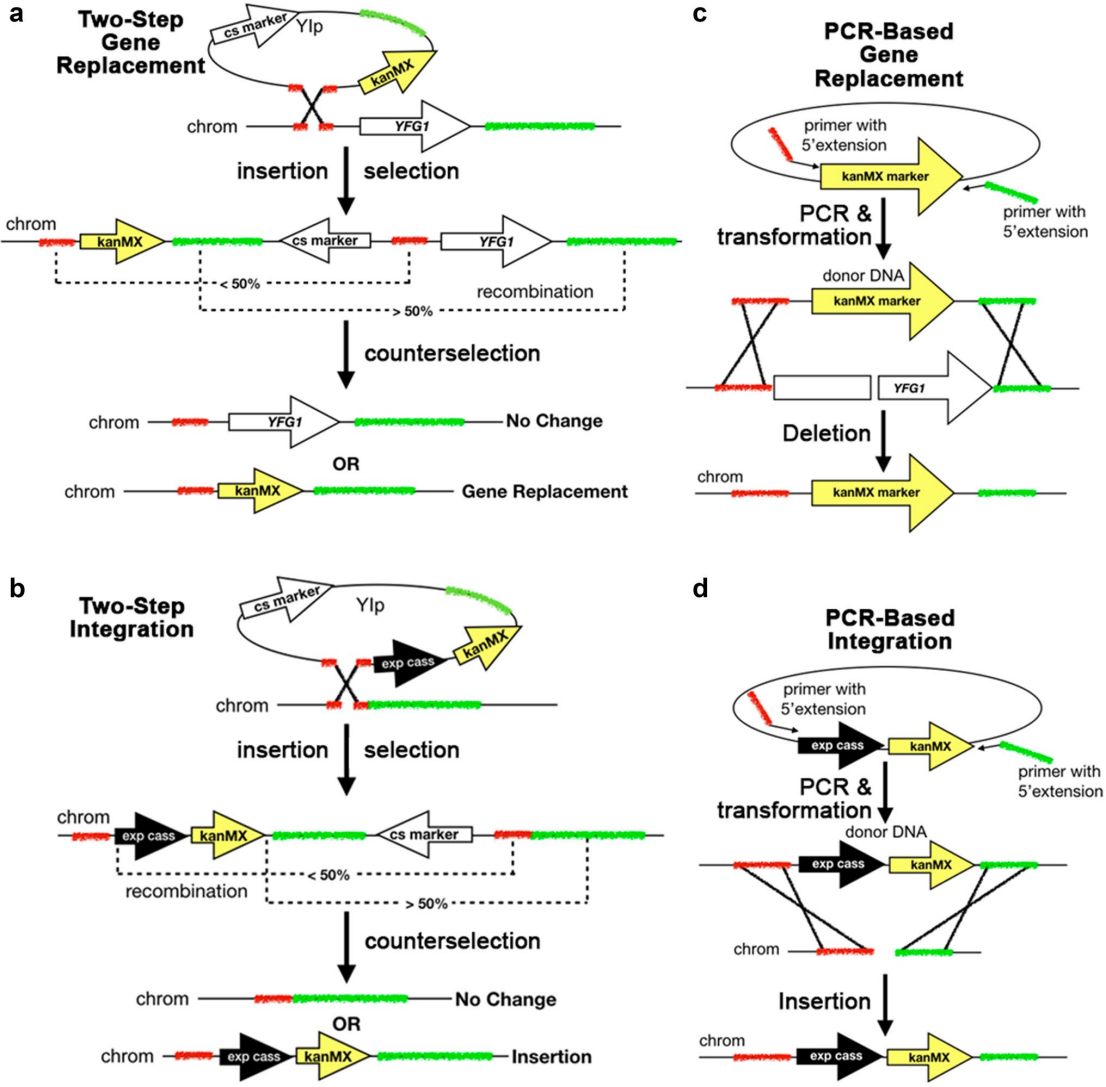
Gene editing approaches to make query mutant strains for GI screens YIp- or PCR-based methods for gene editing introduce donor DNA that serves as a template for homology-directed repair (HDR) when a spontaneous DNA double-strand break (DSB) occurs in the target locus denoted by your favorite gene *yfg1∆*. **a, b** YIp-based gene editing is performed in two steps. In the first step, the YIp linearized at a short cloned region of homology is integrated into the homologous chromosome region (red). The integrated construct generates direct repeats, which can spontaneously recombine and “pop-out” the construct. However, recombination is more likely to occur at the longer repeat region (green), resulting in stable integration of the construct. Recombinants are obtained by counterselection. **a** This Yip is designed to delete *YFG1* and replace it with the *kanR* (*kanMX*) marker. **b** This YIp is designed to integrate an expression cassette (exp cass) in an intergenic chromosomal target. **c, d** PCR-based gene editing uses plasmid cassettes that contain a selectable marker. The target sequences are encoded in 40–60 nts of the PCR primer at 5′ overhangs (red and green). The primers also have ~ 20 nts of homology to the plasmid backbone flanking the cassettes at their 3′ ends (arrows). The same gene-specific primers can be used to insert various types of cassettes at the chromosomal target (red and green). **c** PCR-based gene deletion/replacement with a selectable marker. **d** PCR-based insertion of a marked expression cassette. *kanMX*: G418-resistance marker; *YFG1*: wild-type your favorite gene; *X*: homologous recombination event, *chromI*: chromosomal locus; *exp cass*: expression cassette, *cs marker*: counterselectable marker

In theory, a special class of plasmids called yeast integrating plasmids (YIps) could be used in systematic GI screens (Sikorski and Hieter 1989). YIps do not carry a yeast origin of replication and, consequently, the only way they are stably inherited is by chromosomal integration. Stable integration of YIps is accomplished in two steps using yeast’s very efficient homology-directed repair (HDR) machinery for initial integration of the linearized YIp after transformation, and recombination and counterselection of the plasmid backbone (Storici et al. 2001; Nair and Zhao 2009) (Fig. 2a, b). YIps can be used to introduce gene expression cassettes or gene deletions (Rudolph et al. 1985; Alani et al. 1987; Lopes et al. 1989; Parekh et al. 1996; Voth et al. 2001; Akada et al. 2002; Sakai et al. 2004; Sadowski et al. 2007). However, YIps are only used for systematic GI screens as query plasmids because there are no systematic YIp collections.

### Synthetic genetic arrays (SGA)

Prior to the synthetic genetic array (SGA) method, it was not practical to systematically cross mutants to test GIs. Genetic crosses were performed in small batches and the haploid segregants could only be obtained in one of two ways—tetrad dissection or random spore analysis (Sherman 2002). In tetrad dissection, the sporulation mix, which consists of unsporulated diploid cells and sporulated haploid segregants encased in asci, is mildly digested with a glycosidase to break open the asci. The mix is spread on a plate and the tetrad spores from each ascus (still clustered together) are individually dissected onto different grid positions on the plate and allowed to form clonal colonies. In random spore analysis, sporulation mixes are treated with diethyl ether to kill off all diploids while leaving a small set of resistant viable haploid spores. The treated mix is then spread onto plates to obtain colonies from each surviving spore. In both methods, colonies are assessed for mating type and the presence of each marked mutation to find the desired mutant combinations by replica plating to various types of media.

The power of the SGA approach is that it utilizes a haploid-specific reporter gene to select for haploids of one mating type. This haploid selection reporter uses a *MAT***a**-specific promoter, specifically those driving **a**-factor or α-receptor expression (*MFA1pr* or *STE2pr*, respectively), to drive expression of the *HIS3* auxotrophic marker in *his3* mutant strains. *MAT***a** haploid cells are selected on media lacking histidine. The original reporter, *MFA1pr-HIS3*, showed leaky expression and could recombine with the small *his3∆1* deletion allele present in BY4741/2/3 strains, allowing growth of diploids and *MAT*α strains. An improved version of the haploid reporter uses the more stringent *STE2pr* driving expression of the *Schizosaccharomyces pombe* ortholog *Sphis5* to prevent recombination with *his3∆1* (Daniel et al. 2006). The reporter also knocks out the *CAN1* arginine permease gene for better haploid selection using the toxic arginine analog canavanine. Heterozygous diploid *CAN1*/*can1∆::STE2pr–Sphis5* cells have an intact copy of the permease, which allows canavanine into the cells leading to death. The his + *MAT***a** haploids are *can1∆* and resistant to canavanine. This additional selection prevents the growth of diploids that have undergone gene conversion to *MAT***a**/*MAT***a**, a source of false negatives (Tong and Boone 2007).

A single-query strain carries the LOF or GOF allele of interest plus the haploid reporter gene (Tong and Boone 2006). Introduction of these alleles into the SGA haploid selection strain can be done by crossing an existing mutant strain with the SGA parent strain, or by directly gene editing the SGA parent strain. Gene editing of the query strain is routinely done by YIp-mediated gene deletion/replacement or integration of an overexpression cassette (Fig. 2a, b) or by PCR-mediated gene deletion/replacement or expression cassette integration (Fig. 2c, d), but query strains may also carry episomal plasmids for gene overexpression.

**Fig. 3:**
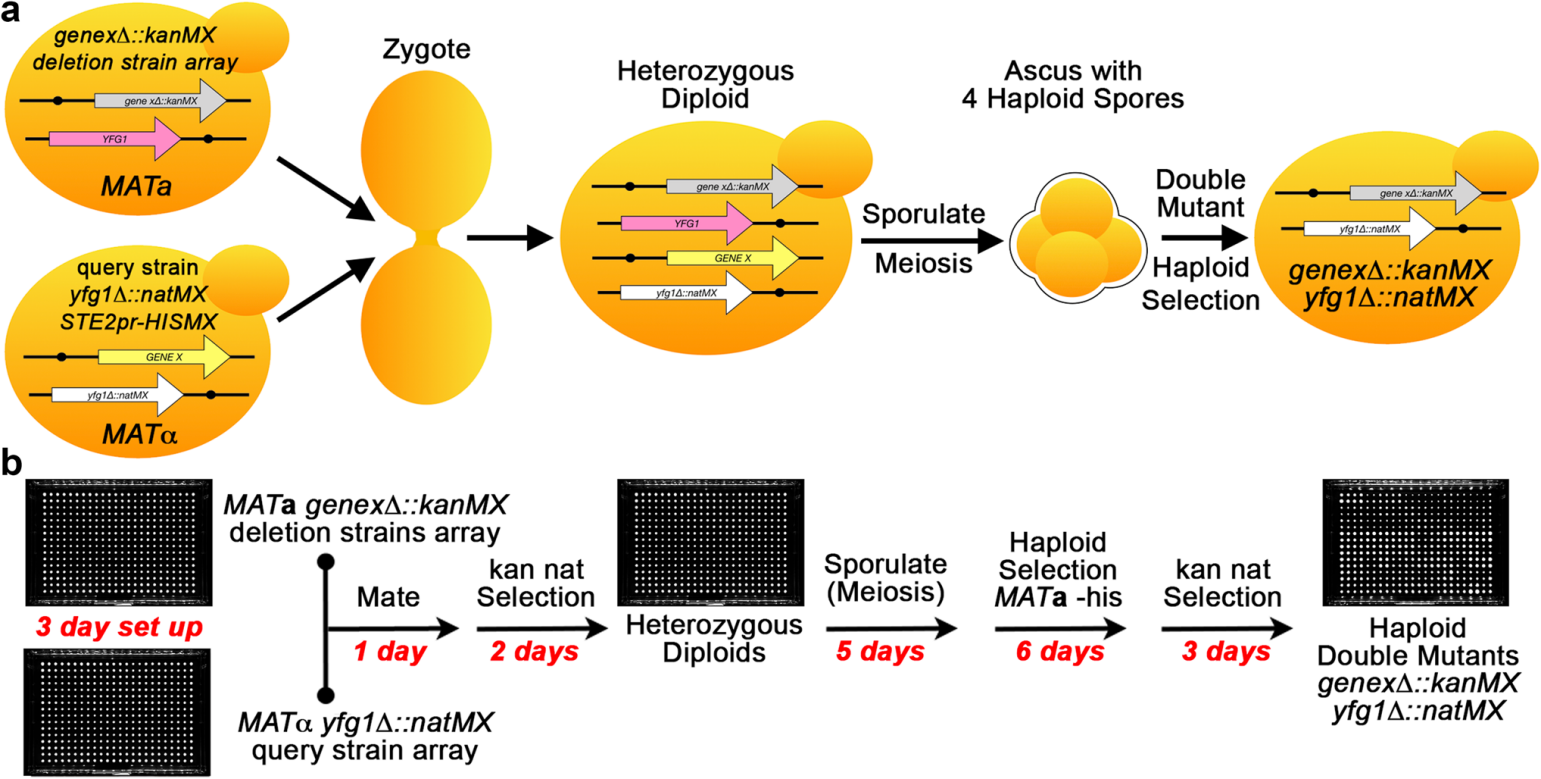
GI screens using SGA. **a** Schematic of the DNA molecules and manipulations resulting in the generation of a double mutant by SGA. **b** The SGA workflow starts with two agar plates of arrayed colonies. Each colony on the arrayed deletion collection plate is a different deletion strain marked with the same selection marker and in the same mating type. The query deletion strain, denoted by your favorite gene *yfg1∆*, is arrayed on a second plate and carries a different selection marker and has the opposite mating type. Colonies from the two parent plates are replica pinned onto the same plate to mix two mutants and allow them to mate and form a heterozygous diploid zygote. The diploids are replica pinned onto sporulation media. The resulting sporulation mix is plated on a series of selection media to obtain haploid strains carrying both parental deletions. Double mutants should be quadruplicated to 384 arrays for statistical measurements of growth. Plates are imaged and colony sizes are measured to determine if two genes interact. Red text indicates how many days are required for each step in the workflow. *yfg1∆::natMX*: query mutation of your favorite gene deleted and replaced with nourseothricin-resistance marker, *genex∆::natMX*: haploid strain collection gene deletion replaced with G418-resistance marker, *STE2pr-HISMX MAT***a**: haploid selection marker that confers growth on media lacking histidine

The query strain is then mated to arrayed colonies from a strain collection (Fig. 3). The query mutation must be linked to a selectable marker that is different from the marker used in the strain collection to allow selection of double mutants. Hundreds of different marked deletion strains can be arrayed on each rectangular agar plate, pinned on top of the query strain to allow each pair of strains to mate, and replica pinned onto double selection media to select for diploids. The diploids are then sporulated, and haploid cells carrying the desired mutant combinations are selected.

The explosion in yeast genetic analyses over the past decade enabled by the SGA method was due to the ability to make thousands of pairwise genetic crosses in parallel (as of this writing, a PubMed search for “synthetic genetic array” and “cerevisiae” produces 120 publications since 2001). At last count, GIs have been tested for over 75% of yeast genes, and a little under 1/3 of all possible ~ 18,000,000 pairwise combinations of annotated yeast genes have been tested (Costanzo et al. 2010).

SGAs were initially used to find synthetic lethal GIs (Tong et al. 2001, 2004; Davierwala et al. 2005; Ooi et al. 2006). Subsequent SGA screens and its variations analyzed more subtle effects of GIs on cellular fitness, including positive (suppression) as well as negative (enhancement) synthetic interactions (Schuldiner et al. 2005; Collins et al. 2010; Costanzo et al. 2010; Baryshnikova et al. 2010; Lindén et al. 2011; Piening et al. 2013; Stundon and Zakian 2015).

SGA has also been used to find synthetic dosage interactions by crossing query strains carrying integrated overexpression cassettes with the deletion collection (Youn et al. 2017), by crossing query strains transformed with OE plasmids (Measday et al. 2005; Douglas et al. 2012) to the deletion strain collection, or by crossing query strains to a collection of strains in which each carries a different OE plasmid (Sopko et al. 2006; Liu et al. 2009; Duffy et al. 2016). Usually, the resulting LOF–GOF double mutants are analyzed as arrays, but they can also be pooled to perform batch analysis of growth by quantifying unique barcodes (by microarray or sequencing) in the arrayed strain collection (Douglas et al. 2012).

Although the SGA approach allows high-throughput genetic crosses, making thousands of strains carrying just two genetic modifications remains a daunting endeavor that requires specialized robotic equipment (about one-third of SGA publications have come from the Boone lab). Although a less expensive semi-automated robotic system has been developed (Rotor HDA by Singer Instruments) to allow SGA automation within the scope of a NIH R01 grant, this tactic also reduces the throughput, adds significant cost to the consumables, and increases the manual labor required. Because of the cost of consumables and extra labor, one should perform a cost–benefit analysis of the Rotor HDA system compared to more automated systems using fewer consumables (e.g., S&P Robotics Inc. systems) using reasonable expectations of the scope and frequency of SGA screens to be performed within 5–10 years.

Another technical limitation of SGA is that it tends to produce false-negative interactions. One reason for this is that haploid deletion strains are under selective pressure to grow faster and often pick up suppressor mutations or become disomic with both the deletion allele and the wild-type gene. For example, when testing purchased haploid deletion strains upon arrival, we found a suppressing mutation in *WHI5* (a transposon insertion) in the *MAT***a***cln3∆* strain (YSC6273-201934719)(unpublished data), and disomy in the *MAT***a***kar9∆* mutant (YSC6273-201917550), which normally has a chromosome segregation defect and is likely aneuploid (unpublished data).

Another technical problem with SGA is cross-contamination from neighboring wells in both the deletion collection and during the SGA process. For example, we found that the *MAT***a***msn5∆* strain (YSC6273-201934942) consisted mostly or entirely of a strain deleted in the adjacent ORF/plate well, *MRX8* (unpublished data). The integrity of the haploid deletion strains is also an issue when transforming a query plasmid into a haploid collection for systematic dosage screens.

Finally, it may be impossible to obtain diploids from strains with strong mating defects or combinations of strains that have bilateral mating defects. However, if one images all diploid selection plates, the missing crosses can be excluded from subsequent SGA analysis of haploid mutant combinations.

### Diploid-based synthetic lethality analysis on microarrays (dSLAM)

**Fig. 4:**
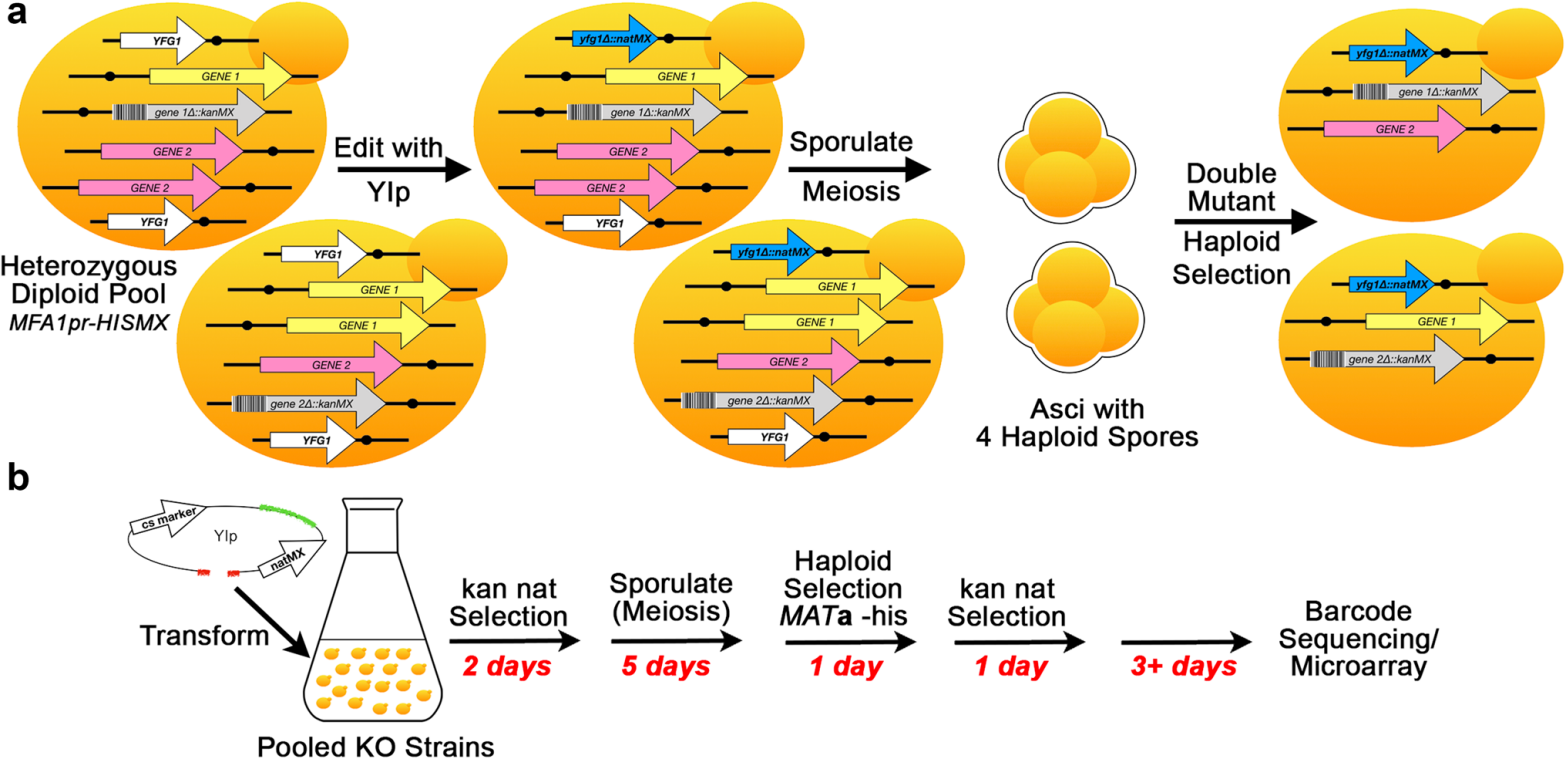
GI screens using dSLAM. **a** Schematic of the DNA molecules and manipulations resulting in the generation of a double mutant by dSLAM. **b** The dSLAM workflow starts with a pool of various (usually the entire collection) competent diploid heterozygous deletion strains that contain the SGA *MAT***a** selection marker. The pooled cells are transformed with a query YIp that will introduce a gene edit such as a deletion (denoted by your favorite gene *yfg1∆*) or an overexpression cassette. The edited cells undergo selection for the edit (nat) and the gene deletions (kan), and are sporulated. After sporulation, *MAT***a** haploid cells are selected on media lacking histidine, and then double mutant haploids are selected. The barcode abundances of the various deletion strain are measured by microarray or sequencing. Depletion of barcodes indicates a negative GI with the query mutation. *gene 1*/*2∆::kanMX*: heterozygous strain collection gene deletions replaced with G418-resistance marker, *YFG1*: wild-type your favorite gene, *yfg1∆::natMX*: query mutation of your favorite gene deleted and replaced with nourseothricin-resistance marker, *MFA1pr-HISMX MAT***a**: haploid selection marker that confers growth on media lacking histidine and marked with G418-resistance marker striped regions—barcodes unique to each gene deletion

Our lab and others have avoided the problem of modifier mutations in the haploid strain collections using fresh haploid deletion strains derived from the heterozygous deletion strain collection. There is little to no selection for modifier mutations in the heterozygous diploids. However, this approach requires sporulation and dissection or random spore analysis of haploid spores to derive strain arrays for SGA and is not practical for genome-wide SGA screens. In a streamlined variation of this approach, diploid-based synthetic lethality analysis on microarrays (dSLAM) introduces the SGA haploid reporter construct into pooled strains of the heterozygous diploid deletion collection (Pan et al. 2004) (Fig. 4). The pooled diploid strains are then transformed with a YIp to delete or overexpress a gene. The pool of double mutants is selected and then sporulated. After sporulation haploid double mutant progeny are selected and the pool is analyzed by microarray (or sequencing) for the relative abundances of unique barcodes associated with each deletion in the strain collection compared to the control strains, in which *YFG1* is replaced by wild-type *YFG1::URA3*. Because strain identification is performed by microarray or sequencing of each gene deletion’s unique barcode, strain identity cannot be misattributed as it can with potentially cross-contaminated plate arrays. In addition, the various heterozygous diploid strains largely grow at the same rate, avoiding the bias in gene representation seen in pooled haploid deletion strains.

To our knowledge, dSLAM has been used only by the group that developed the method. The lack of widespread adoption of this approach is likely due to the technical complexity of barcode quantification by microarray hybridization or sequencing. Both methods require very careful bioinformatics analysis of the raw data that are beyond the capabilities of labs that do not routinely use such genomic methods and do not have a bioinformatician on staff. Nevertheless, dSLAM has been used to screen for synthetic lethal, dosage synthetic lethal, dosage suppression, and synthetic suppression interactions (Pan et al. 2004; Kim et al. 2009).

Although dSLAM seeks to avoid many of the limitations of SGA, it is very likely to lose completeness of genome coverage due to lack of transformation of random deletion strains in the pool of strains. More than 5% of genes will be excluded from any given analysis because the deletion strain did not integrate the query mutation or the marked wild-type query allele for the control (Pan et al. 2004). Moreover, some of the diploid deletion barcodes have been mutated during strain construction and do not hybridize well to microarrays, which are designed for the haploid strain barcodes (Pan et al. 2004). Using sequencing approaches to quantify the barcodes (amplifying from flanking universal primer tags) should solve this problem.

### The Green Monster

**Fig. 5:**
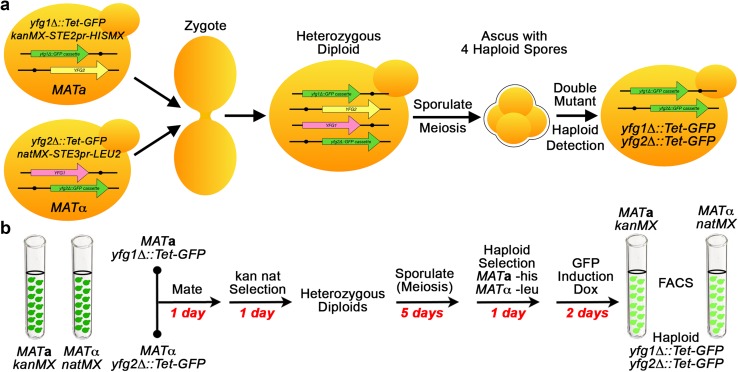
GI screens using green monster. **a** Schematic of the DNA molecules and manipulations resulting in the generation of a double mutant by Green Monster. **b** The Green Monster workflow starts with various strains from *MAT***a** and *MAT*α haploid deletion collections in which the *kanMX* marker is replaced with a Tet-induced GFP expression cassette (denoted by your favorite gene *yfg1∆* and *yfg2∆*). The *MAT***a** strains also carry a GM*Toolkit*-**a** (*kanMX-STE2pr-HISMX*) and the *MAT*α strains carry a GM*Toolkit*-α (*natMX-STE3pr-LEU2*), both of which are derived from the SGA haploid selection reporter. These strains are randomly mated in bulk, and diploids are grown under double selection conditions. The resulting heterozygous diploids are sporulated and haploids of both mating types are selected. FACS analysis sorts cells that have twice the GFP intensity of the single mutants and, therefore, carry two deletions. The entire culture can be re-mated without FACS analysis to make mutants that carry 3, or more gene deletions. Red text indicates how many days are required for each step in the workflow. *yfg1*/*2∆::Tet-GFP*: query deletions of your favorite genes deleted and replaced with Tet-inducible GFP, *kanMX*-*STE2pr-HISMX MAT***a**: haploid selection marker that confers growth on media lacking histidine and marked with G418-resistance marker, *natMX*-*STE3pr-LEU2 MAT*α: haploid selection marker that confers growth on media lacking leucine and marked with nourseothricin-resistance marker

An approach to multi-mutant strain construction, called the Green Monster, is essentially SGA performed with a pooled collection of strains (Fig. 5). Different ORFs are replaced with a green fluorescent protein (GFP) expression cassette in strains carrying the SGA haploid selection markers (Suzuki et al. 2011). Using GFP, the Green Monster method bypasses the need to mark each gene deletion with a different selectable marker. The authors found a near-linear correlation between fluorescence intensity and the number of deletions. Combinations of deletions are made by mating and sporulating pools of deletion/GFP strains and then passing them through a fluorescence activated cell sorter to find progressively brighter cells. One may also perform multiple rounds of mating, sporulation, and haploid selection and only sort cells after numerous rounds to save time. Performing all steps in liquid cultures saves time and cost of media, and, because strains bearing multiple deletions from previous rounds can mate, fewer rounds of crosses are needed to obtain cells carrying all of the gene deletions in the pool compared to SGA.

Suzuki et al. (2011) used the Green Monster method to make a mutant in which 16 different ABC transporter genes associated with multi-drug resistance are deleted. The mutant’s drug sensitivity spectrum was analyzed and compared to the wild-type parent and a mutant carrying deletions of eight different ABC transporters. To the best of our knowledge, this study is the only use of the Green Monster for genetic analysis.

Although this method is much faster than SGA, it still requires multiple rounds of crosses and also careful quantification of GFP by fluorescence-activated cell sorting (FACS). Furthermore, finding specific intermediate strains requires considerable screening of sorted cells and it is difficult to determine if the inability to find certain combinations of mutations is due to negative GIs or technical problems, especially when using large numbers of deletion strains. The authors obtained intermediate strains by crossing pools of subsets of all the deletion strains. The requirement for single-cell sorting and screening to find intermediate mutants and for precise fluorescence measurements by FACS has probably limited adoption of this method, although few researchers have attempted to make large numbers of strains carrying 4 or more mutant alleles.

## Genetic screens in the age of CRISPR/Cas

**Fig. 6:**
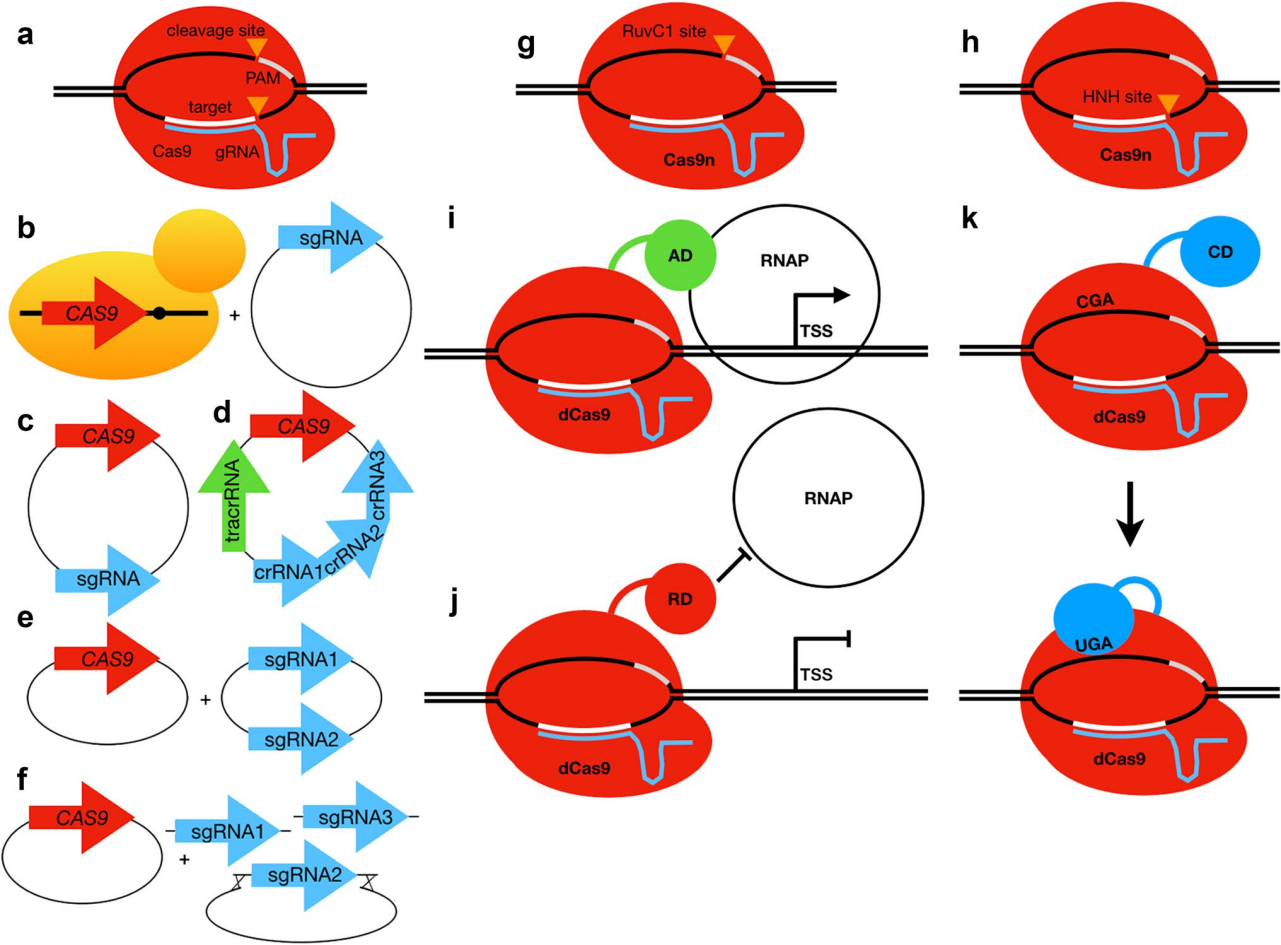
Using CRISPR/Cas for genome editing. **a** Schematic showing how Cas9-gRNA generate DSBs at programmed target sites. **b**–**f** Different expression systems for CRISPR/Cas9 editing in yeast. **b** A yeast strain expressing inducible Cas9 from an integrated cassette requires a plasmid expressing sgRNA. **c** A CRISPR/Cas expression system requiring only one plasmid expressing both Cas9 and sgRNA. **d** A multiplex CRISPR/Cas system expressing Cas9, a tracrRNA, and a CRISPR/Cas array containing different crRNAs. **e** A multiplex CRISPR/Cas system expressing Cas9 and two or more sgRNAs on separate plasmids. **f** A multiplex CRISPR/Cas system expressing Cas9 from one plasmid, and using in vivo gap repair of a selectable plasmid by linear sgRNA cassettes to generate the sgRNA expression plasmid. **g** Schematic showing how nCas9 (D10A mutation in the RuvC1 endonuclease active site) generates a nick on the opposite strand of the target site. **h** Schematic showing how nCas9 (H840A mutation in the HNH endonuclease active site) generates a nick on the same strand of the target site. **i** Schematic showing how catalytically inactive dCas9 can be fused to an activation domain (AD) to recruit RNA polymerase (RNAP) and promote transcription of the target gene. **j** Schematic showing how catalytically inactive dCas9 can be fused to a repressor domain (RD) to inhibit RNA polymerase (RNAP) and prevent transcription of the target gene. **k** Schematic showing how catalytically inactive dCas9 can be fused to cytidine deaminase (CD) to convert a cytosine to a uracil. A round of repair and replication either converts the U back to C, or converts the U to a T to generate a TGA STOP codon. *PAM*: protospacer adjacent motif, *gRNA*: guide RNA including the Cas-interacting stem-loop structure, *tracrRNA*: gRNA portion that hybridizes with crRNA and contains stem-loop, *crRNA*: gRNA portion that hybridizes with crRNA and contains DNA targeting sequence, *sgRNA*: a gRNA that combines the tracrRNA and crRNA in a single RNA molecule, *AD*: activation domain, *RD*: repressor domain, *CD*: cytidine deaminase, *RNAP*: RNA polymerase II, *TSS*: transcription start site

The efficiency of gene editing by HDR with donor DNA is vastly improved when a DSB is introduced at the target locus using a sequence-specific endonuclease (Guha and Edgell 2017). Because of the ease of programming its sequence specificity, the most versatile endonuclease-based genome editing system is CRISPR/Cas (Fig. 6a). There are several excellent reviews on the discovery and mechanism of CRISPR/Cas (Lander 2016; Stovicek et al. 2017; Raschmanová et al. 2018).

### Yeast CRISPR/Cas systems

The CRISPR/Cas system was first adapted for gene editing in mammalian cells (Jinek et al. 2013; Cong et al. 2013; Mali et al. 2013), soon followed by applications in many other organisms, including budding yeast (Dicarlo et al. 2013). In the case of mammalian cells, DSBs generated by endonucleases are usually repaired by non-homologous end joining (NHEJ) even in the presence of donor DNA for HDR (Guha and Edgell 2017). Mammalian cell researchers have used a variety of methods to increase the frequency of HDR (Kooistra et al. 2004; Pöggeler and Kück 2006; Choquer et al. 2008; Fox et al. 2009; Certo et al. 2012; Delacôte et al. 2013; Verbeke et al. 2013; Ran et al. 2013; He et al. 2014; Lin et al. 2014; Schwartz et al. 2017). However, in *S. cerevisiae*, efforts to increase HDR over NHEJ are not necessary. In fact, NHEJ in yeast is so rare that only HDR is used for editing (although CRISPR/Cas is useful for mechanistic studies of NHEJ) (Gallagher and Haber 2018; Lemos et al. 2018).

There are over two dozen CRISPR/Cas toolkits for *S. cerevisiae*, most of which have been described in detail by Stovicek et al. (2017) and Raschmanová et al. (2018). Most CRISPR/Cas editing tools combine the naturally separate CRISPR RNA (crRNA) and trans-activating CRISPR RNA (tracrRNA) in a hybrid small guide RNA (sgRNA). The generic term gRNA refers to either tracrRNA-crRNA, crRNA, or sgRNA. The main differences between the various yeast CRISPR/Cas systems are their plasmid structures.

There are a few systems that integrate the Cas9 expression construct into the yeast genome (Fig. 6b) (Horwitz et al. 2015; Mans et al. 2015; Vanegas et al. 2017), but most systems express Cas9 from a plasmid (Fig. 6c–f). Integration of Cas9 has several advantages. It circumvents the requirement for transformation with a Cas9 expression plasmid (leaving more available markers for multiplexing) and subsequent counterselection of the plasmid to remove Cas9. The integrated Cas9 cassettes are also inducible. However, to remove the potential influence of the integrated Cas9 on strain phenotype (due to leaky basal expression), the Cas9 cassette should be removed by targeting it with a further gRNA and replacing the endogenous locus (Finnigan and Thorner 2016). In some yeast CRISPR/Cas systems, the Cas9 and gRNA expression cassettes are on the same plasmid, which improves the efficiency of transformation (Fig. 6c, d). However, single plasmid systems are incompatible with some of the gRNA cloning approaches. For example, because Cas9 is large and generating a Cas9-gRNA plasmid by PCR amplification can be challenging (Ryan and Cate 2014). In addition, editing efficiencies are much higher if Cas9 is expressed before introduction of the gRNA (Walter et al. 2016), making CRISPR/Cas editing inefficient when using a single Cas-gRNA plasmid. Finally, making many different mutant combinations of genes by multiplexing could be more easily accomplished by having separate expression cassettes for each gene (Fig. 6e, f).

There is significant variability in efficiency between different loci and gRNAs, probably due to differences in gRNA secondary structure that affect gRNA binding to its target and/or nucleosome occupancy at or near the target sequence (Smith et al. 2016; Horlbeck et al. 2016; Thyme et al. 2016). Because of this unpredictable variability, several gRNAs must be tested for each target gene (Ryan et al. 2014; Shalem et al. 2014; Wang et al. 2014; Koike-Yusa et al. 2014; Zhou et al. 2014; Konermann et al. 2015; Chen et al. 2017; Smith et al. 2017; Sadhu et al. 2018). It is generally presumed that as more gRNAs are tested in yeast, empirical or mechanistic design rules will improve. An additional and important consideration in designing gRNAs is the potential for off-target DNA binding due to sequence similarities. Although off-target mutations when using CRISPR to edit genes are of debatable concern in higher eukaryotes (Fu et al. 2013; Cho et al. 2014; Wang et al. 2015, 2018a, b; Iyer et al. 2015, 2018; Muhammad et al. 2016; Zhang et al. 2018), off-target mutations are considered to be unlikely in yeast due to their small genomes (making it easier to find stringent target sequences) and low error-prone NHEJ activity (Ryan et al. 2014; Jakočiunas et al. 2015a). Consistently, deep sequencing of CRISPR edited strains showed no off-target mutations (Jakočiunas et al. 2015a).

Nevertheless, off-target editing is likely to depend largely on the gRNA sequence and Cas9 activity. Reducing the likelihood of off-target mutations can be accomplished with numerous online gRNA design tools (reviewed in Stovicek et al. 2017). One can also ensure target specificity using Cas9n, a nickase in which one of the two endonuclease active sites is mutated (Fig. 6g, h), with two closely spaced gRNAs. Off-target DNA nicking by a single Cas9n will be quickly repaired, but only the combination of two close nicks on opposite strands will produce a (staggered) DSB.

CRISPR/Cas has seen only limited use for systematic functional genomics or genetic screens in yeast (Smith et al. 2016, 2017; Chen et al. 2017; Roy et al. 2018; Sadhu et al. 2018; Guo et al. 2018), largely because there were already many different genomic mutant collections when CRISPR/Cas gene-editing was developed. Nevertheless, CRISPR/Cas has been used to probe gene function in ways that current strain collections cannot, including examining ORFs that are not mutated in any strain collection. In addition, CRISPR/Cas editing can be markerless and scarless, avoiding possible phenotypic effects of selectable markers and exogenous DNA (Acton et al. 2017; Elison and Acar 2018). When making markerless/scarless edits, if the desired mutation(s) does not occur in an available gRNA target, the donor DNA must include multiple silent mutations in the PAM and/or target sequence to prevent cyclic re-cutting (Horwitz et al. 2015). Of course, in non-coding regions, neutral mutations cannot be predicted. One can avoid making silent mutations using a two-step process in which one first replaces the PAM-target sequence with heterologous “stuffer” donor sequence (Biot-Pelletier and Martin 2016; Elison and Acar 2018). The stuffer can then be replaced with donor DNA encoding only the desired point mutation by exchanging the original gRNA with a gRNA targeting the stuffer. The second donor DNA can reconstitute the original PAM-target sequence without consequence.

### Yeast CRISPR/Cas libraries

Several yeast CRISPR/Cas libraries have been designed for generating LOF mutants. In the library produced by Sadhu et al. (2018), gRNAs are paired with donor DNA designed to introduce STOP codons. This library was designed to target all annotated essential genes in yeast consisting of ~ 10,000 gRNA-donor plasmids targeting different portions of ~ 1000 essential genes (Sadhu et al. 2018). The gRNAs were designed to replace the cas-binding protospacer adjacent motif (PAM) sites (NGG) in the target DNA with stop codons (TGA or TAG). This functional genomic screen showed that many essential genes could tolerate C-terminal truncations. Guo et al. (2018) similarly used a paired gRNA-donor CRISPR/Cas library to make small START codon deletions in a set of ~ 300 verified ORFs and an equal number of unverified small ORFs (smORFs) to determine which of these ORFs (which are not deleted in the KO collection) are functional (Guo et al. 2018). Roy et al. (2018) used a gRNA-donor CRISPR/Cas library to individually introduce ~ 35,000 natural sequence variants (SNPs and indels) from a wine yeast into the S288C lab strain (Roy et al. 2018). The sequence variants were selected based on whether they altered a PAM or nearby sequences that would be included in the gRNA sequence.

Rather than developing gRNA libraries, a number of groups have developed donor DNA libraries for massive editing of a handful of genomic loci with just a few gRNAs. For instance, Kuivanen et al. developed a synthetic promoter library for multiplexed promoter replacement (Kuivanen et al. 2018). Si et al. (2017) developed a cDNA library that expresses sense or anti-sense RNA depending on the orientation of the ORF in a standard expression cassette. This library included > 90% of annotated genes. They then used CRISPR/Cas9 to integrate the pooled library into multiple ∂ sites in a yeast strain that expresses RNAi machinery (Dicer, Ago2 and TRBP; *S. cerevisiae* does not naturally perform RNAi) (Si et al. 2017). Sense strand cassettes generated GOF alleles while anti-sense cassettes generated LOF alleles. They used this approach to perform multiplex integrations of random GOF and LOF alleles and screened the pooled transformants for improvements in several metabolic traits.

Instead of directly editing genes to analyze LOF mutations, CRISPR/Cas can also be used to modulate gene expression to find GOF or LOF phenotypes. One approach is to use dCas9, a catalytically inactive cas9 mutant, fused to a transcriptional activator or repressor domain to produce GOF or LOF conditions—CRISPRa and CRISPRi, respectively (Fig. 6i, j) (Larson et al. 2013; Perez-Pinera et al. 2013; Gilbert et al. 2013, 2014; Maeder et al. 2013; Konermann et al. 2015). Smith et al. (2016) developed a pooled plasmid library of 989 gRNAs against the 5′UTRs of 20 genes to perform CRISPRi for a chemical genetics screen (Smith et al. 2016). This group subsequently developed an arrayed strain collection in which a CRISPRi gRNA library and inducible dCas9 were chromosomally integrated to knock-down 1357 essential yeast genes in 3,832 strains (Smith et al. 2017). In contrast, Chen et al. (2017) searched for GOF interactions by developing a yeast CRISPRa pooled plasmid library consisting of ~ 10^7^ random gRNAs, which they used in conjunction with a dCas9-VP64 synthetic transcriptional activator (Chen et al. 2017). They used this gRNA library to perform a screen for sgRNAs that suppressed lethality of human α-synuclein overexpression.

So far, the studies of Si et al. (2017) and Chen et al. (2017) are the only uses of CRISPR/Cas to perform systematic GI screens, and both employed CRISPRi/a without mutating genes. There are several limitations to adoption of CRISPR/Cas editing to perform systematic GI screens. Most important is the lack of genome-wide gRNA plasmid libraries suitable for GI screens. LOF gRNA libraries that could be used to transform query mutant strains for GI screens include the premature termination codon library of Sadhu et al. (2018) and the small deletion library of Guo et al. (2018). However, both libraries focus on ORFs not represented in the haploid deletion strain collections namely essential genes and smORFs, respectively. The only genome-wide (and random) gRNA library currently available is the GOF (CRISPRa) library of Chen et al. (2017). Second, all current systematic gRNA plasmid libraries are designed to generate genetic variation in pooled strains, requiring one to select for, or screen variants within a heterogenous population of transformants (Smith et al. 2016; Chen et al. 2017; Sadhu et al. 2018; Guo et al. 2018). Although, identifying genetic changes associated with phenotypes can be determined by sequencing the gRNA expression cassettes or associated barcodes, it is not possible to consistently find and measure the fitness of strains exhibiting subtle GIs in these pools. Finally, with the exception of the Chen et al. (2017) CRISPRa gRNA library, current gRNA plasmid libraries pair the gRNA with donor DNA on the same plasmid, locking in the type of gene edit.

Crucially, in all of these studies, transformations were performed in a manner to ensure that most cells receive only one plasmid, preventing multiplexing. Even if transformations were performed with high titers of plasmid, there is no way to select for rare multi-mutant transformants in such pools as the donor DNA is unmarked. In the Chen et al. (2017) study, multiple genes were down-regulated using a single gRNA—in essence mirroring multi-gene LOF GIs. Whether the gRNA was able to directly target each of these genes, or whether it knocked down a common transcription factor for these genes was not determined. They also found suppressed strains carrying multiple gRNAs, but did not study these further (Chen et al. 2017).

None of the CRISPR/Cas libraries discussed above have been deposited in commercial or public repositories, but some should be available by request.

### Hypothetical workflow comparison of SGA and CRISPR/Cas

**Fig. 7:**
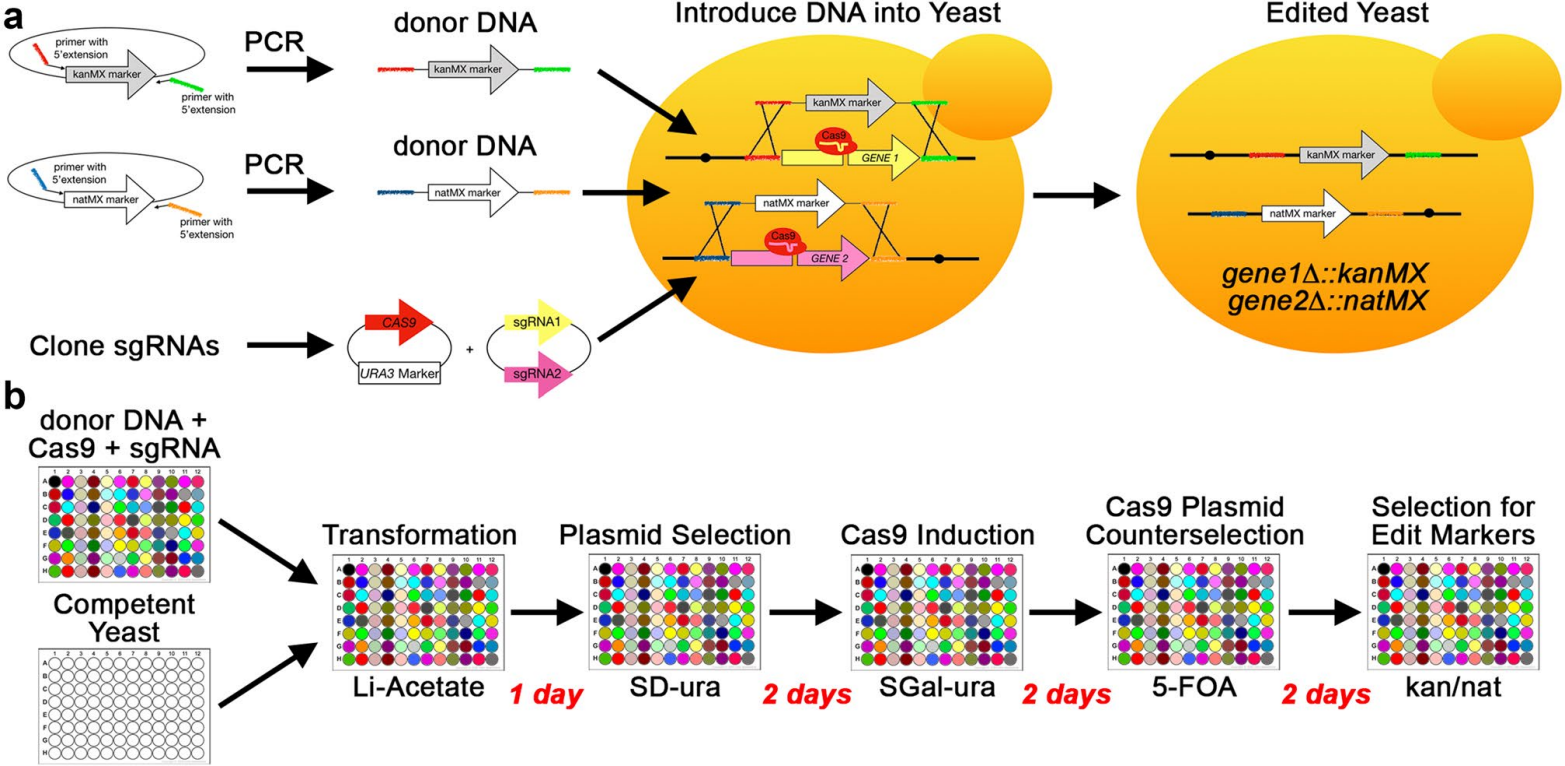
GI screens using multiplexed CRISPR/Cas gene editing. **a** Schematic of the DNA molecules and manipulations resulting in the generation of a double mutant using multiplexed CRISPR/Cas to generated two gene deletions replaced with two different selectable markers. **b** High-throughput workflow to generate multiplex edits. All CRISPR/Cas manipulations are performed in 96-well plates. In this example, all wells carry competent cells of the same strain (wild-type or mutant), but two different sgRNA-Donor plasmids are combined in each well. All wells also receive the same Cas9 expression plasmid. After selection of triply transformed cells, Cas9 expression is induced for multiple generations. Counterselection of the Cas9 plasmid shuts off gene editing, and doubly edited cells are selected (each edit has a different selectable marker). The red text indicates how many days are required for each step in the workflow. *kanMX*: G418-resistance marker, *natMX*: nourseothricin-resistance marker; colored wells indicate that the contents of each well are different

Regardless of the availability of CRISPR/Cas plasmid libraries, if one is interested in a few dozen genes, using CRISPR/Cas to make thousands of possible combinations of edited versions of these genes is very feasible from a cost perspective, and could be accomplished much faster than could be done by SGA (Fig. 7).

For example, if one would like to test all combinations of two to six deletions of a set of 10 genes (837 possible combinations) to find synthetic suppressor/enhancer interactions, one would have to perform 5 consecutive SGA crosses to first make double mutants, then triple mutants, etc. These iterations with single mutants are necessary because the products of SGA crosses cannot be combined, for example, to make quadruple mutants from two sets of double mutants made by SGA of single mutants (Tong and Boone 2006).

CRISPR/Cas does not require crossing strains and/or sporulation of diploids and, therefore, is much faster than SGA, dSLAM, and Green Monster approaches. To make the same mutants by CRISPR/Cas, one would start by designing, cloning, and testing 2–3 gRNAs and 10 donor DNAs, one for each of the 10 genes to determine which gRNAs are best able to perform the desired edits. Multiple gRNAs are tested for each target gene because of the aforementioned problems in predicting gRNA efficiency. Using CRISPR/Cas with validated gRNAs, one could potentially make all 837 mutant strains by performing transformations in 96-well plates with the appropriate combination of gRNA plasmids and donor DNA in each well (Fig. 7b). Although markerless edits would be possible, the screening process would not easily translate to a high-throughput workflow, especially when dealing with editing efficiencies of < 25% for 4 + multiplexed edits. Therefore, this process would benefit from the use of a different selectable marker for each gene in the desired combination.

Using CRISPR, a separate strain does not have to be made for each deletion and marker pair to facilitate crosses. Instead, each deletion is encoded in a donor DNA-gRNA pair that can be combined with other donor DNAs and gRNAs to multiplex gene deletions. These donor DNAs are easily generated using a single primer pair with homology arms specific to each target gene to PCR amplify a set of marker cassettes using common primer binding sites (Fig. 2a). Nevertheless, planning which gRNAs and donor DNAs go into each well and minimizing the number of CRISPR/Cas donor DNAs required to make the desired genetic combinations could benefit from automated workflow design (Pratapa et al. 2018).

An alternative application of CRISPR/Cas that would bypass the requirement for plasmid and donor DNA transformation employs a gene drive CRISPR/Cas cassette. In this approach, the cassette replaces an ORF in a haploid strain and consists of inducible Cas9 and sgRNA expression constructs (DiCarlo et al. 2015; Roggenkamp et al. 2017; Vaschetto 2018). The sgRNA targets the wild-type sequence of the gene deleted by the gene drive cassette. Mating the query strain to an array of collection strains such as the OE collection, produces a heterozygous diploid. Subsequent Cas9 induction will cut and convert the wild-type gene into the gene drive cassette, thus generating a homozygous diploid that can be assessed for synthetic dosage interactions. The cassette could also include a recessive allele of the target gene rather than a deletion (Roggenkamp et al. 2017). This method bypasses the need to sporulate and select for haploids, thus shaving a few weeks off the standard SGA approach, and has been used in *Candida albicans* to generate arrays of a few hundred double mutants targeting genes with related functions (Shapiro et al. 2018).

## Discussion and future work

Despite the small size and simplicity of the *S. cerevisiae* genome, systematically interrogating its pairwise GI spectrum has occupied yeast geneticists for nearly two decades, and is ongoing (Costanzo et al. 2010). It is clear from this massive effort that there exists a significant level of robustness in yeast cells, partly due to network architecture resulting in compensatory feedback mechanisms and alternative pathways, and partly due to functional redundancy between genes (Gu et al. 2003; Guan et al. 2007; Bajić et al. 2014; Bauer et al. 2015; Bader et al. 2015; Cohen et al. 2016; Patra et al. 2017; Ishikawa et al. 2017; Veitia 2017). To better understand genetic robustness and genotype–phenotype relationships, it is imperative that we map multi-gene interactions. Efforts to do so are in their infancy (Haber et al. 2013; Kuzmin et al. 2018), largely because performing such systematic screens is technically demanding with current methods. So far, the multiplexing capabilities of CRISPR/Cas have been exploited mostly by metabolic engineers and synthetic biologists constructing complex genetic circuits and biosynthetic pathways in a handful of strains (Shao et al. 2009; Mikkelsen et al. 2012; Hasunuma et al. 2014; Ryan et al. 2014; Stovicek et al. 2015; Tsai et al. 2015; Jakočiunas et al. 2015b; Ronda et al. 2015; Walter et al. 2016; Jessop-Fabre et al. 2016; Shi et al. 2016; Garst et al. 2017; Kuivanen et al. 2018). However, geneticists could also take advantage of the multiplexing capabilities of CRISPR/Cas to perform systematic GI screens to generate large numbers of strains carrying three or more alleles.

We have described a variety of methods that have been utilized to perform systematic GI screens. In Table 1, we compare and contrast the main features of these approaches. Only three methods—SGA, the Green Monster, and CRISPR/Cas editing—are capable of systematically generating strains carrying three or more mutant alleles. However, it is unclear if the generation of strains carrying three or more mutations is even practical by iterative SGA. Because of the high frequency of false negatives, the SGA approach requires validation of the progeny strains before using them in further crosses. Studies that have generated triple mutants by SGA have all started with double mutant query strains (Haber et al. 2013; Kuzmin et al. 2018).

**Table 1:** Comparison of GI screen methods

Method	Available systematic strain collections	Available systematic plasmid libraries	Time to combine 2 alleles^a^	Time to combine 3 alleles^a^	Types of GIs^b^	Selection method	Plasmids required
OE plasmid	Yes^c^	Yes^g^	3 days	N/A	DS, DE	2 markers	Episomal
SGA	Yes^d^	Yes^g^	17 days	34 days	DS, DE, SS, SE	2 + markers	No^i^
dSLAM	Yes^d^	No	11 days	N/A	DS, DE, SS, SE	2 markers	YIp
Green Monster	No^e^	N/A	11 days	22 days	SS, SE	GFP intensity	No
CRISPR/Cas	Yes^f^	Yes^h^	7 days	7 days	DS, DE, SS, SE	2 + markers	Episomal

^a^Starting with single-gene alleles. Not including genotyping of strains. Approximate time given in days

^b^*DS* dosage suppression, *DE* dosage enhancement including synthetic dosage lethality, *SS* synthetic suppression, *SE* synthetic enhancement including synthetic lethality

^c^Sopko et al. (2006), Douglas et al. (2012), Youn et al. (2017), also deletion strain collections can be used in conjunction with a query plasmid

^d^Giaever et al. (2002)

^e^Giaever et al. (2002) the haploid KO collection is used as the base strain, but the *kanMX* cassettes must be converted to the Green Monster GFP cassette

^f^Smith et al. (2017)

^g^Zhu et al. (2001), Gelperin et al. (2005), Moriya et al. (2006), Hu et al. (2007), Magtanong et al. (2011)

^h^Smith et al. (2016), Chen et al. (2017), Guo et al. (2018), Sadhu et al. (2018)

^i^For dosage screens, the query strain can carry an episomal OE plasmid or an integrated OE cassette

Moreover, the reagents do not currently exist for the Green Monster to perform systematic screens, which would require the replacement of the *kanMX* cassette with the GFP cassette in at least a large proportion of the ~ 5000 haploid KO strains. Finally, current CRISPR/Cas gRNA libraries do not include most of the non-essential ORFs and these libraries are not well-suited to multiplexing because their donor DNAs are unmarked. To use unmarked alleles in a high-throughput genetic screen would require multiplex editing efficiencies of nearly 100% (in practice < 75% for 3 edits), particularly when one is searching for negative GIs, as cells that do not incorporate the desired edits would have growth advantages.

One might wonder what benefit genome-scale yeast CRISPR/Cas libraries could have over current yeast strain collections? Even when analyzing singly edited strains, the use of CRISPR/Cas libraries have produced more specific genetic modifications than current collections, resulting in new insights into gene structure and function (Sadhu et al. 2018; Guo et al. 2018). In addition, even simple LOF genome-scale CRISPR/Cas libraries have the potential to quickly assess highly combinatorial GI networks by editing existing systematic mutant strain collections. However, to expand their combinatorial potential, future CRISPR/Cas library designs will have to enhance multiplexing.

Although the gRNAs are the smallest component of CRISPR/Cas systems, the cost of automation and DNA reagents to make genome-scale gRNA plasmid collections is likely prohibitive for most labs. As we mentioned above, gRNA libraries must include several gRNAs for each target, making the size of genome-wide gRNA libraries very large (tens or hundreds of thousands), even for a small genome like yeast. Smith et al. (2017) addressed this issue by developing a more cost-effective method of generating genome-wide sequence-verified gRNA libraries (several thousand dollars in reagents and a few months of labor), and applied this method to the construction of a collection of CRISPRi strains allowing inducible knockdowns of most essential genes (Smith et al. 2017). In addition to the cost of gRNA oligonucleotide libraries, cloning of the gRNAs into expression vectors is a labor-intensive process, even with streamlined methods of cloning [reviewed in (Stovicek et al. 2017)].

Making genome-wide CRISPR/Cas libraries in yeast could also be simplified by using Cas9 derivatives that do not use HDR and donor DNA. We already discussed how CRISPRa/i can produce knockdown or dosage effects by directly influencing expression of the target genes without needing to also introduce a donor DNA (Gilbert et al. 2013; Smith et al. 2016, 2017; Chen et al. 2017). However, positioning of the dCas9 fusion protein is crucial to transcriptional activity. Therefore, the gRNA libraries for CRISPRi/a tend to be even larger than the knock-out libraries since each gene is targeted by more gRNAs (e.g., 10 per target gene). Another CRISPR/Cas method that does not require donor DNA uses a catalytically inactive dCas9 fused to cytidine deaminase to change targeted CAG, CGA, CAA or TGG to the STOP codons TAG, TGA or TAA, respectively, after a round of repair and replication (Fig. 6k) (Nishida et al. 2016; Komor et al. 2016; Kuscu et al. 2017; Billon et al. 2017). The mismatch repair machinery of the cell has a 50–50 chance of repairing the U instead of the G, but by making a nick in the G-containing strand using an Cas9n-cytidine deaminase fusion, one can trick the cell into saving the modified base. Unfortunately, the deaminase tends to also modify off-target cytidines, so future improvements to this method will couple the deaminase activity to PAM-target binding (Komor et al. 2016).

Because efficient gene editing in *S. cerevisiae* has been possible for more than 20 years, CRISPR/Cas has not been as widely adopted as in other model systems. Consequently, there are not yet many yeast CRISPR/Cas libraries, and these are pooled, requiring microarrays or sequencing to identify mutants that grow better or worse. In contrast, because yeast plasmid libraries and strain collections are arrayed, the function of each gene can be assessed for more complex phenotypes than relative growth/fitness in a population. Although they are more labor-intensive than pooled libraries, arrayed CRISPR/Cas libraries would allow one to perform more diverse phenotypic tests, including high content imaging (Henser-Brownhill et al. 2017; de Groot et al. 2018). Arrayed libraries also ensure that no genomic targets are missed, a distinct possibility when using pooled plasmid libraries that require occasional amplification. The generation of arrayed yeast CRISPR/Cas libraries is an inevitable next step.

Almost all of the current yeast strain systematic collections have been generated by gene editing using transformation with PCR products amplified from plasmids carrying cassettes of selectable markers alone or combined with protein-tagging or promoter-replacement sequences (Längle-Rouault and Jacobs 1995; Longtine et al. 1998; Baker Brachmann et al. 1998; Bähler et al. 1998; Goldstein and McCusker 1999; Gueldener 2002; Janke et al. 2004; Hentges et al. 2005; Akada et al. 2006). CRISPR/Cas could be used to modify existing genomic strain collections using a single gRNA and, in most cases, a single-donor DNA, in a manner akin to integrating a target sequence into various chromosomal loci and using a single gRNA to edit at all locations (Finnigan and Thorner 2016; Giersch and Finnigan 2017), except that the target sequences are already integrated in the yeast strain collection.

Using CRISPR/Cas to edit deletion strain collections, one could remove the *kanMX* marker from the deletion strains and replace it with markerless donor DNA fragments homologous to the two ends of the *kanMX* cassette. Although other methods have been used to make markerless deletions from the deletion collection, they also remove the deletion barcodes (Carvalho et al. 2013; Soreanu et al. 2018). Similarly, one could swap the *kanMX* marker from the deletion strains with other MX markers. Marker swapping methods using plasmids or PCR cassettes have been in use for over a decade (Alani et al. 1987; Longtine et al. 1998; Baker Brachmann et al. 1998; Goldstein and McCusker 1999; Voth et al. 2003). However, none have been used to systematically swap the markers of genomic collections. Marker-swapped collections would be useful for SGA, facilitating comprehensive SGA screens and also making the production of three or more edited combinations easier. One could use CRISPR/Cas to replace deletion strain markers with the GFP expression cassette used for the Green Monster method allowing for systematic construction of multi-deletion strains faster than possible using SGA. A systematic collection of gene drive deletion strains could be generated by replacing the *kanMX* cassette with gene drive cassettes containing an sgRNA specific to the wild-type copy of each deleted gene. This collection could be used to make double mutants using an SGA approach to cross query gene drive strains to the gene drive collection and inducing Cas9 to generate homozygous double mutants. Finally, one could introduce fiducial markers at any chromosomal location to study chromatin-chromosome dynamics. For example, using CRISPR/Cas9, Soreanu et al. (2018) replaced a *natMX* gene deletion with a TetO array in cells expressing TetR-tdTomato (Soreanu et al. 2018). The same approach could be used to systematically mark positions along all chromosomes using the deletion collection, or using a gRNA library paired with a dCas9-fluorescent protein (Chen and Huang 2014).

Using CRISPR/Cas to edit the GFP or TAP strain collection, one could replace the C-terminal fusion cassette with modern variants of GFP, other fluorescent protein derivatives, or other protein fusion tags. Replacement of the old cassettes could simultaneously remove the marker. Roggenkamp et al. (2017) tested a CRISPR/Cas method to do just this, but so far this approach has only been used to modify a few strains at a time (Roggenkamp et al. 2017). One could also replace the C-terminal tag cassette with a markerless MS2 or PP7 cassette for mRNA tagging. However, in this approach, the homology arms of the donor DNA would have to be specific to each ORF to assure that the mRNA tag alone is integrated between the STOP codon and 3′UTR.

Despite the versatility and ease of gene editing in yeast, the generation of genome-wide strain collections is a costly and time-consuming process usually performed by a consortium of labs. Consequently, yeast cell biologists and geneticists have had to rely on strain collections that are sometimes not optimal for their needs. Moreover, current high-throughput methods of generating multi-mutant strains rely on slow methods that all require the production of multi-mutant diploids and sporulation to obtain multi-mutant haploids. CRISPR/Cas has the potential both to rapidly enhance our current genomic strain collections and to systematically generate genome-wide 3-way or even higher dimensional GI maps.
